# Sex differences in variability across timescales in BALB/c mice

**DOI:** 10.1186/s13293-016-0125-3

**Published:** 2017-02-09

**Authors:** Benjamin L. Smarr, Azure D. Grant, Irving Zucker, Brian J. Prendergast, Lance J. Kriegsfeld

**Affiliations:** 1 Department of Psychology, University of California, Berkeley, CA 94720 USA; 2 Department of Integrative Biology, University of California, Berkeley, CA USA; 3 Department of Psychology, University of Chicago, Chicago, IL USA; 4 The Helen Wills Neuroscience Institute, University of California, Berkeley, CA 94720 USA

**Keywords:** Temporal structure, Longitudinal data, Biological rhythms, Ovulation, Estrous cycles, Wavelets, Time series analysis

## Abstract

**Background:**

Females are markedly underinvestigated in the biological and behavioral sciences due to the presumption that cyclic hormonal changes across the ovulatory cycle introduce excess variability to measures of interest in comparison to males. However, recent analyses indicate that male and female mice and rats exhibit comparable variability across numerous physiological and behavioral measures, even when the stage of the estrous cycle is not considered. Hormonal changes across the ovulatory cycle likely contribute cyclic, intra-individual variability in females, but the source(s) of male variability has, to our knowledge, not been investigated. It is unclear whether male variability, like that of females, is temporally structured and, therefore, quantifiable and predictable. Finally, whether males and females exhibit variability on similar time scales has not been explored.

**Methods:**

These questions were addressed by collecting chronic, high temporal resolution locomotor activity (LA) and core body temperature (CBT) data from male and female BALB/c mice.

**Results:**

Contrary to expectation, males are more variable than females over the course of the day (diel variability) and exhibit higher intra-individual daily range than females in both LA and CBT. Between mice of a given sex, variability is comparable for LA but the inter-individual daily range in CBT is greater for males. To identify potential rhythmic processes contributing to these sex differences, we employed wavelet transformations across a range of periodicities (1–39 h).

**Conclusions:**

Although variability in circadian power is comparable between the sexes for both LA and CBT, infradian variability is greater in females and ultradian variability is greater in males. Thus, exclusion of female mice from studies because of estrous cycle variability may increase variance in investigations where only male measures are collected over a span of several hours and limit generalization of findings from males to females.

## Background

When exploring questions in fields other than reproductive biology, it is generally assumed that sex differences in physiology are negligible [1]. Additionally, female mice of various strains are known to show changes in physiology and behavior across the estrous cycle [2–4]. These factors have created a perception that females are more variable and therefore more difficult to study than males, leading researchers to neglect females to focus on males with findings generalized to females of the same species [5]. This male bias has not only led to false assumptions regarding female physiology and behavior but has also been harmful. For example, cancer chronotherapies developed in studies of men revealed that treatments at a particular circadian phase increased survival rates but that the same treatment regimen significantly decreased female survival [6, 7]. Until recently, whether females are actually more variable than males has not been empirically investigated [8]. In a meta-analysis of 293 articles, the variance of behavioral, morphological, physiological, and molecular measures was not significantly greater in female than male mice for any parameter and was substantially greater in males for several traits [9]. A similar analysis of rats (311 articles) yielded similar results [10].

While one source of female variability likely results from hormonal fluctuations during the ovulatory cycle, the source and pattern of male variability has not been systematically characterized. We sought to examine sex differences in two widely used and widely applicable modalities: locomotor activity (LA) and core body temperature (CBT). LA and CBT are commonly used as proxies for physiological and neurological outputs in biology. In rodents, for example, daily changes in LA and CBT are associated with the progression of the estrous cycle [11–14]. The progressive delay in the offset of daily LA from the day of estrus in Syrian hamsters [14] and a corresponding delay in the daily decline of CBT on the day of estrus in C57 mice [13] point to the utility of LA and CBT in tracking physiologically relevant events and sources of variation.

Stereotyped changes in LA or CBT over both the day and the estrous cycle demonstrate that these modalities are structured across multiple timescales and that this temporal structure explains much of the variability in these data. To investigate potential sex differences at different timescales, we used wavelet transformation (WT) of LA and CBT data. Wavelets possess position, frequency, and amplitude information and are ideally suited for analysis of overlapping cycles occurring in the same data at different time scales. WTs have been successfully applied to studies of circadian biology to capture changes in power by periodicity across time [15–17], underscoring the efficacy of this approach. By uncovering sex differences and temporal structure in variability across timescales, studies can be designed to effectively consider these variables. Additionally, as LA and CBT effectively mirror underlying physiological change, identifying predictable patterns of variability sets the stage for further exploration of rhythmic patterns in underlying biological systems [18–22].

## Methods

### Animals

Data were analyzed from 13 male and 13 female BALB/c mice (>60 days of age) in accordance with protocols approved by the Animal Care and Use Committee at UC Berkeley and in conformance with principles enunciated in the NIH Guide for the care and use of laboratory animals. Mice were housed under an LD 12:12 photocycle with ad libitum access to water and chow. Light onset and offset occurred at 0600 and 1800 h, respectively. Humidity and temperature were held constant at 40% and 21 °C, respectively.

### Core body temperature and locomotor activity data collection

Data were gathered with Mini Mitter G2 E-Mitter implants that chronically record LA and CBT (Starr Life Sciences Co., Oakmont, PA). G2 E-Mitters were implanted in the intraperitoneal cavity (under isoflurane anesthesia, with analgesia achieved by subcutaneous injections of 0.03 mg/kg buprenorphine in saline, administered every 12 h for 2 days after surgery). E-Mitters were sutured to the ventral muscle wall to maintain consistent core temperature measurements. Recordings began immediately, but data collected for the first week post-surgery were not included in analyses. Recordings were continuous and stored in 1-min bins. Implants were placed in 7- to 10-week-old mice that were handled once per week during recordings (at the time of cage changes) but otherwise left undisturbed in single housing. Light intensity during the photo- and scotophases were ~400 lux white light and <1 lux red light, respectively.

### Data analysis

Twelve days of data from each mouse were selected for analysis. In females, these days were aligned to a day of estrus, defined by the presence of an extended period of maximum CBT [13]. Thus, the 12 days encompassed three consecutive 4-day estrous cycles, with the first, fifth, and ninth days demonstrating an estrus-like profile characterized by a prolonged plateau of peak CBT, as previously reported [13]. For males, 12 consecutive days of data were chosen. For CBT, values below 35 °C were set to 35 °C to remove the result of a few rare device malfunctions and all values more than three standard deviations from the mean were set to three standard deviations from the mean. For LA, the correction to three standard deviations was only applied in the positive direction so that erroneously high values were corrected while activity counts of “0” were not inflated. The output from the G2 implant is in the form of degree Celsius and activity counts per unit time (events per 1 min).

Daily range for each modality was defined as (max–min) per mouse per day. Median 4-day windows (i.e., cycles in females) were generated for each animal by taking the average of the three repeated cycles, followed by taking the average of these 4-day windows across individuals of the same sex. Inter-animal variability was defined as the population range for each modality’s daily range.

Data were analyzed and plotted using Matlab 2015b and 2016a in conjunction with in-house code for wavelet decomposition modified from the “Jlab” toolbox and from code developed by Prof. Tanya Leise [15], using the Morse wavelet (Morse parameters of *β* = 5 and *γ* = 3 that describe the frequencies of the two waves superimposed to create the wavelet) (see [23] for further description). Briefly, whereas Fourier transforms allow transformation of a signal into frequency space without temporal position (i.e., using sine wave components with infinite length), wavelets are constructed with amplitude diminishing to 0 in both directions from center. This property permits frequency strength calculation at a given position. Wavelets can assume many functions (shapes, e.g., Mexican hat, square wave, Morse); here, we use a Morse wavelet with relatively low number of oscillations (defined by *β* and *γ*), similar to wavelets used in previous circadian applications [15, 24]. This low number of oscillations enhances detection of contrast and transitions. Additional values of *β* (3–8) and *γ* (2–5) did not alter the findings. Because WTs exhibit artifacts at the edges of the data being transformed, WTs were performed on 14-day windows for each animal, with the 12 days previously analyzed, buffered by 1 day preceding and 1 day following. To avoid erroneous edge effects, only the WT of the 12 days analyzed previously were analyzed further. Periods of 1 to 39 h were assessed. For statistical comparisons of populations, Wilcoxon rank sum tests were used to avoid assumptions of normality for any distribution; note that degrees of freedom are not used in Wilcoxon rank sum tests. Non-parametric Kruskal-Wallis tests were used instead of ANOVAs for the same reason; for all Kruskal-Wallis tests, *χ* ^2^ and *p* values are listed in the text and all such comparisons have the same *N*/group, and so the same degrees of freedom (df = *N*/group − 1 = 12). For quantification of spectral differences, WT spectra were isolated in two 2-h bands; circadian periodicity power was defined as the max power per minute within the 23–25-h band; ultradian periodicity power was defined as the max power per minute in the 1–3-h band. The latter band was chosen because this band corresponded with the daily ultradian peak power observed in ultradian rhythms (URs) across physiological systems [21, 22, 24–31]. A color map for wavelets was developed by our group to provide red-green colorblind-compliant uniform contrast across the range of data, with the exception of the two extremes (highest and lowest 10%), which are brightened to highlight extreme high and low values. Figures were formatted in Microsoft PowerPoint 2013 and Adobe Photoshop CS6.

## Results

**Fig. 1:**
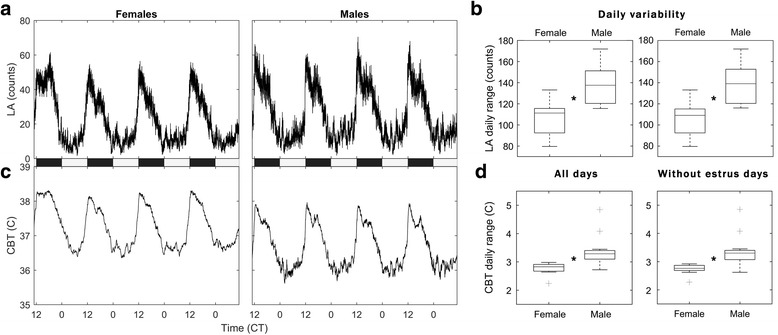
Males exhibit more daily variability than females. Median 4-day plots of locomotor activity (LA; **a**) and core body temperature (CBT; **c**) for all females and males, from 12 days of 13 mice each; the *light*:*dark cycle* is shown along the abscissa. Day 1 is the day of estrus for females, with arbitrarily selected four consecutive days shown for males for comparison. Females show expected 4-day cycles of LA and CBT that are absent in males. Box plots of the daily variability comparing females to males (**b**, **d**) reveal that males have a higher intra-animal daily range in LA and CBT. *Plus symbol* indicates outliers. *Asterisk* indicates a significant sex difference (without estrus, LA: *p* < 1 × 10^−4^; CBT: *p* = 1 × 10^−4^; with estrus, LA: *p* < 1 × 10^−4^; CBT: *p* < 1 × 10^−4^). Males also exhibit a wider population range in CBT, but the population range in LA is comparable for both sexes. The two plots on the *right* indicate that, whereas estrus changes the structure of both variables across the day, it does not increase the overall range of either variable in females (all days including estrus, LA: *p* = 1 × 10^−4^; CBT: *p* < 1 × 10^−4^). Males appear to show higher amplitude, high frequency oscillation in the inactive (*light*) phase

As expected, females exhibited a 4-day cycle in LA and CBT (Fig. 1a, c). Females show a prolonged peak of daily LA and CBT on the day of estrus, as described previously [13, 14], while males do not exhibit an obvious 4-day cycle. Within-animal daily range was significantly greater in males for CBT (Fig. 1d; *p* < 1 × 10^−4^) and LA (Fig. 1b; *p* < 1 × 10^−4^). Between-animal variability was greater for males in CBT and comparable to females in LA. (Fig. 1b, d; bars extending from each box; CBT: *R*_male_ = 2.12, *R*_fem_ = 0.75; LA: *R*_male_ = 56.34, *R*_fem_ = 53.49). To assess whether or not estrus increased the inter- or intra-individual range of female LA or CBT, we reanalyzed these daily ranges excluding days of estrus (i.e., for parity, excluding days 1, 5, and 9 from the analysis in both males and females). As predicted, estrus did not substantially change either the inter- or intra-individual ranges in these parameters, nor did it enhance the differences across sexes observed with all days included (Fig. 1b, d, left vs. right; CBT: *p* < 1 × 10^−4^ without estrus vs. 1 × 10^−4^ with all days; LA: *p* = 1 × 10^−4^ without estrus vs. *p* = 1 × 10^−4^). This is not to imply that estrus does not represent a source of infradian variance in females; rather, that the day of estrus does not increase the daily variability of female LA or CBT. Instead, estrus manifests as a change in the shape of LA and CBT across the day, with a longer plateau of peak temperature and activity during the active phase than on non-estrous days.

**Fig. 2:**
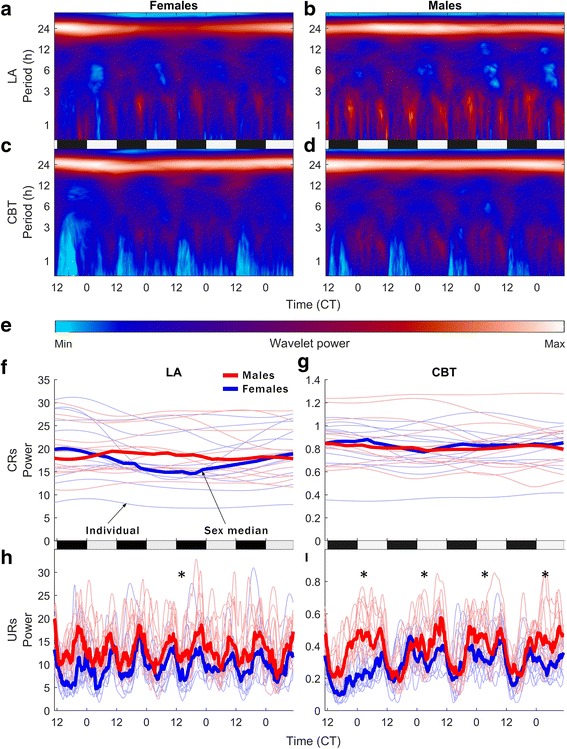
Median wavelet transforms (WTs; **a**–**d**) of the data that comprise the average depicted in Fig. 1 indicate changes in rhythmic power (**e**) across periodicities ranging from 1 to 39 h (*log y-axis*). Day 1 is the day of estrus for females (**a**, **c**) and an arbitrary four consecutive days chosen for males (**b**, **d**). Females show an effect of day in ultradian CBT, the depression of which identifies estrus (*χ* ^2^ = 13.52, *p* < 5 × 10^−3^). Males do not show an effect of day but exhibit greater power in high frequency (1–3-h periodicity) URs in LA (*χ* ^2^ = 34.36, *p* = 1 × 10^−8^). Quantification of individual and median circadian power (CRs, 23−25-h) (**f**, **g**) and ultradian power (URs, 1−3-h) (**h**, **i**). For CBT, male UR power is greater only during the inactive phase (**i**). *Asterisk* indicates a significant sex difference. Both sexes show an effect of time of day for URs in CBT, but males have nearly twice the ultradian change of females (*χ* ^2^ = 54.83, *p* < 1 × 10^−12^ for males; *χ* ^2^ = 30.61, *p* < 1 × 10^−7^ for females). No sex difference is detectable in the circadian range (23–25-h)

WT analysis reveals sex differences in LA and CBT at the ultradian and infradian, but not circadian periodicity. Neither sex exhibits an effect of day at the circadian periodicity (23–25 h) for either LA (*χ* ^2^ = 3.64, *p* > 0.05, *χ* ^2^ = 0.37, *p* > 0.05 for females and males, respectively) or CBT (*χ* ^2^ = 0.85, *p* > 0.05, *χ* ^2^ = 0.68, *p* > 0.05 for females and males, respectively) (Fig. 2a–g). Female infradian rhythms manifest as a significant reduction in 1–3-h ultradian power specifically on the day of estrus as compared to the ultradian power in any of the subsequent 3 days (Fig. 2h, i) (*χ* ^2^ = 13.52, *p* < 1 × 10^−3^). Males have significantly higher power 1–3-h URs in LA than females (Fig. 2h) (*χ* ^2^ = 34.36, *p* < 1 × 10^−8^). Furthermore, males exhibit a change from dark to light phase CBT UR power nearly twice that of females (Fig. 2i) (*χ* ^2^ = 54.83, *p* < 1 × 10^−12^ for males; *χ* ^2^ = 30.61, *p* < 1 × 10^−7^ for females). Females are more variable than males on the infradian scale, but males are more variable than females on an ultradian scale.

## Discussion

Female rodents have long been discounted as study subjects because estrous cycles are assumed to generate greater variability across traits than is seen in males. Until recently, substantive data to support or refute this conjecture were lacking. Two meta-analyses now have demonstrated for mice [9] and rats [10] that females tested at random stages of the estrous cycle are no more variable than males for numerous traits, consistent with genetic profiling arrays [32]; similar data have been obtained for unstaged human subjects [28]. The source of variability in males over the course of 4 days corresponding to the female estrous cycle has not been examined prior to the present study. Contrary to earlier assumptions, we found that median circadian power is comparable between the sexes. Whereas females exhibit infradian rhythms associated with the estrous cycle, males show significantly greater UR power than females, suggesting that differences in timescales of biological rhythms may help explain overall similarity in variability between the sexes [9, 10]. Stated differently, females vary across infradian cycles of 4 days but males are more variable than females within a single day. These findings underscore that males should not necessarily be favored over females in rodent studies and point to sex differences across timescales as worthy of explicit exploration in systems of interest.

To understand the significance of sex differences in ultradian power, it is necessary to understand the physiological dynamics underlying CBT temporal structure. URs have not been extensively investigated, but several candidate systems both exhibit URs in the 1–3-h range and modulate CBT. These systems include the hypothalamo-pituitary-gonadal axis [13, 18–20, 33], the hypothalamo-pituitary-adrenal axis [21, 24–29, 34, 35], and the suprachiasmatic nucleus [30]. The central dopaminergic axis exhibits URs in the 1–3-h range [31] and may influence CBT as well through modulation of activity and appetitive behaviors, like eating and drinking. If each of these systems independently modifies CBT, one would expect to see a peak of ultradian power for each system. However, our data show a relative consolidation of ultradian frequencies, consistent with a framework in which these distinct physiological systems belong to a network of coupled oscillators. Oscillators that couple eventually drive each other to synchrony [36]. Physiologically, some evidence already exists for coupling across these systems [37]. For example, luteinizing hormone (LH), a pituitary peptide well known for regulating reproductive axis functioning, also affects steroid hormone production in the adrenals [38]. In turn, adrenal steroids can feed back to the brain to inhibit LH [39, 40]. However, the physical extent and the temporal persistence of this hypothetical-coupled network within an individual have not been systematically studied.

As relationships connecting LA and CBT rhythms with more difficult-to-measure endpoints are described, this knowledge can be used to design studies that eliminate time-consuming, disruptive assays such as repeated blood collection [41] or to estimate outcome measures in between collection points to add greater effective temporal resolution. If CBT rhythms reflect changes in several physiological systems, then males may show larger variability than females across those systems at the ultradian time scale (e.g., higher amplitude change in URs of sex-hormone release, feeding). Whether the present findings generalize to rats or other rodents that undergo estrous cycles is presently unknown and subject to experimental verification. A range of factors affecting temporal structure in addition to sex (e.g., age, strain, species) warrant exploration using similar approaches so that a maximum variance in physiological measures can be accounted for.

## Conclusions

This study confirms that male BALB/c mice show greater variability across the day, while females show an infradian rhythm that males do not. Our finding of significant sex differences in ultradian power suggests that inclusion of female mice is advantageous where observations are made over the course of several hours—in contrast to current practice, which generally excludes female mice from many experiments [9]; because male ultradian variability exceeds that of females, females are more likely to provide consistent results when measures are obtained over the course of several hours. Finally, whereas the mechanisms that generate URs in CBT remain unknown, the difference in UR power points to basic differences in male and female physiology. Investigating URs in both sexes in tandem may provide novel insights into sex differences in physiology and behavior. In light of the many sex differences in human biology, studying both sexes will increase the translational impact of mouse research, helping to avoid inappropriate generalization of findings from males to females.

## Abbreviations

CBT: Core body temperature
LA: Locomotor activity
UR: Ultradian rhythm
WT: Wavelet transformation
